# Diagnosis of brain death in Brazil

**DOI:** 10.5935/0103-507X.20190050

**Published:** 2019

**Authors:** Glauco Adrieno Westphal, Viviane Cordeiro Veiga, Cristiano Augusto Franke

**Affiliations:** 1 Central Estadual de Transplantes de Santa Catarina, Secretaria de Estado da Saúde de Santa Catarina - Florianópolis (SC), Brasil.; 2 Unidade de Terapia Intensiva, Hospital Municipal São José - Joinville (SC), Brasil.; 3 Unidade de Terapia Intensiva Neurológica, Hospital BP - A Beneficência Portuguesa de São Paulo - São Paulo (SP), Brasil.; 4 Serviço de Medicina Intensiva, Hospital de Clínicas de Porto Alegre, Universidade Federal do Rio Grande do Sul - Porto Alegre (RS), Brasil.; 5 Unidade de Terapia Intensiva do Trauma, Hospital de Pronto Socorro de Porto Alegre - Porto Alegre (RS), Brasil.

**Keywords:** Brain death/diagnosis, Brazil

## Abstract

Brain death, defined as the complete and irreversible loss of brain functions, has a history that is linked to the emergence of intensive care units and the advancement of artificial ventilatory support. In Brazil, by federal law, the criteria for the diagnosis of brain death have been defined by the Federal Council of Medicine since 1997 and apply to the entire Brazilian territory. Resolution 2,173/2017 of the Federal Council of Medicine updated the criteria for diagnosing brain death. These changes include the following: the requirement for the patient to meet specific physiological prerequisites and for the physician to provide optimized care to the patient before starting the procedures for diagnosing brain death and to perform complementary tests, as well as the need for specific training for physicians who make this diagnosis. Other changes include the reduction of the time interval between the two clinical examinations, the possibility of continuing procedures in the presence of unilateral ear or eye injury, the performance of a single apnea test and the creation of a statement of brain death determination that includes the recording of all procedures in a single document. This document, despite the controversy surrounding it, increases the safety necessary when establishing a diagnosis of such importance and has positive implications that extend beyond the patient and the physician to reach the entire health system.

## INTRODUCTION

The first description of the clinical and pathological findings that characterize brain death (BD) was made by Mollaret and Goullon in 1959. The main characteristics observed in the group studied were deep coma, lack of spontaneous breathing and isoelectric electroencephalogram.^(1)^

In Brazil,^(2)^ law 9,434, from February 4, 1997, determined that it was the responsibility of the Federal Council of Medicine (*Conselho Federal de Medicina* - CFM) to establish BD diagnostic criteria and, through Resolution 1,480/97,^(2,3)^ determined that BD would be considered in cases of neurological conditions of known cause and defined as an irreversible process. To make this diagnosis, two clinical examinations and one complementary test would be necessary.

Decree 9,175, from October 18, 2017, reinforced that the CFM was in charge of determining the BD criteria.^(4)^ Since then, CFM Resolution 2,173, from November 23, 2017, has defined BD as the complete and irreversible loss of brain functions, defined by the cessation of cortical and brainstem activities.^(5)^

## CHANGES IN THE METHODOLOGY FOR DIAGNOSING BRAIN DEATH

CFM Resolution 2,173 brought changes to the methodology for diagnosing BD, which are highlighted in bold throughout the text and in table 1.^(5)^

**Table 1:** Main changes in the methodology for the diagnosis of brain death

1.	Participation of the neurologist ceases to be mandatory
2.	Physicians should be specifically trained to diagnose BD
3.	Determination of a minimum duration of observation and treatment before beginning the determination of BD
4.	Compliance with physiological prerequisites for the diagnosis of BD
5.	Reduction of the time interval between the two clinical examinations
6.	Possibility of clinical examination in cases of unilateral anatomical lesion of the eyes or ears
7.	Performance of a single apnea test
8.	Pretest blood gas analysis, ideally with PaO_2_ ≥ 200mmHg and PaCO_2_ between 35 and 45mmHg
9.	Possibility of performing the apnea test with the use of CPAP
10.	Adequate communication with family members before and during the entire BD diagnostic process
11.	Interruption of life support when organ donation is not feasible
12.	New Statement of Determination of BD to be completed by all physicians involved in the diagnosis

BD - brain death; PaO_2_ - partial pressure of oxygen; PaCO_2_ - partial pressure of carbon dioxide; CPAP - continuous positive airway pressure.

According to the first article of the resolution, the procedures for determining BD should be started **in all patients with suspected BD** - those with deep coma, lack of supraspinal reflexes, and persistent apnea - as **long as they meet the prerequisites described in table 2**.

**Table 2:** Mandatory procedures for determining brain death

**A. Communication of the suspicion of BD to relatives**		
- Relatives should be informed about the suspicion of brain death and the stages of its determination		
- Family members should receive updated information at each step of the BD determination process.		
**B. Notification of the BD**		
- Notify the State Transplant Center that the determination of BD has been initiated		
**C. Prerequisites to be met at the beginning of and during the BD determination procedure**		
- Presence of brain injury of known cause, irreversible and capable of causing BD.		
- Absence of treatable factors that may confound the diagnosis of BD (e.g., sedatives)		
- Treatment and observation at a hospital for a minimum of 6 hours. This period of observation and treatment must be at least 24 hours in cases of hypoxic-ischemic encephalopathy or after the rewarming phase of therapeutic hypothermia		
- Body temperature> 35ºC, SatO_2_ > 94% and blood pressure according to age group:		
**Age group**	**SBP (mmHg)**	**MAP (mmHg)**
≥ 16 years	100	65
7 - 16 years noninclusive	90	65
2 - 7 years noninclusive	85	62
5 months - 2 years noninclusive	80	60
Up to 5 months noninclusive	60	43
**D. Two clinical examinations that detect signs compatible with BD**		
- Deep coma		
- Absence of brain stem reflexes		
pupillary light		
corneal-palpebral		
oculo-cephalic		
vestibulo-ocular		
cough		
**Age**	**Minimum time interval between the two examinations**	
7 full days (full-term infant) up to 2 months	24 hours	
2 - 24 months noninclusive	12 hours	
Older than 2 years	1 hour	
**E. Apnea test **		
- lack of spontaneous breathing movements after cessation of ventilation during maximal stimulation of the respiratory center with documentation of PaCO_2_ > 55mmHg		
**F. Complementary tests**		
- Electroencephalogram		
- Cerebral angiography		
- Transcranial Doppler		
- Cerebral scintigraphy		
**G. Conduct after the determination of brain death**		
- Mandatory notification of BD to the State Transplant Center		
- Completion of the DC. If death is due to external cause, the DC should be completed by the coroner		
- Report death to family members as soon as it is determined		
- Any mention of organ donation should occur only after communication of death		
- Withdrawal of life support in cases where organ donation is not feasible		

BD - brain death; SatO_2_ - arterial oxygen saturation; SBP - systolic blood pressure; MAP - mean arterial pressure; PaCO_2_ - partial pressure of carbon dioxide; DC - death certificate.

- There is a need for two clinical examinations performed by two different physicians to make this diagnosis, but **the involvement of a neurologist is no longer mandatory**.^(4,5)^ Now, in addition to neurologists or neurosurgeons (adult or pediatric), intensivists (adult or pediatric) and emergency physicians can also, when properly trained, establish the diagnosis of BD. In case of unavailability of these specialists, the examination may be performed by another duly trained physician. That is, in practice, there is no specialty prerequisite, as **long as the doctor is properly trained**. Another important change is the **reduction of the time interval between the first and second clinical examinations according to age group** (Table 2).
- The neurological signs compatible with BD are (1) deep coma and (2) absence of brainstem reflexes, to be tested in the rostrocaudal sequence (Table 2). One of the changes in the new resolution is that it allows the performance of clinical examination in cases of **congenital or acquired unilateral anatomical lesions of the eyes or ears**. However, **bilateral eye and ear injury or suspected or confirmed spine injury prevent the determination of BD**.^(5)^
- The apnea test remains mandatory and is now performed **only once**, after the **prerequisites are met**, as described in the step-by-step procedure in table 3. **A pretest blood gas analysis is mandatory** after 10 minutes of preoxygenation with 100% fraction of inspired oxygen (FiO_2_). According to the original wording of resolution 2.173/2017, **arterial blood pressure (PaO_2_) ≥ 200mmHg and partial carbon dioxide pressure (PaCO_2_) between 35 and 45mmHg were also required** before discontinuation of mechanical ventilation. However, an amendment published on November 23, 2018, indicated that these blood gas values **should ideally be obtained, but are not necessary**.^(5)^
  **Table 3:** Procedures for safe performance of the apnea test

**A. Preparation of the apnea test**
- Monitor and stabilize the patient
- Body temperature > 35ºC, SatO_2_ > 94% and blood pressure according to age group
- Absence of treatable factors that may interfere with the breathing movements (e.g., sedatives)
- Preoxygenation with 100% FiO_2_ for 10 minutes
- Initial blood gas analysis, **ideally** obtaining PaO_2_ ≥ 200mmHg and PaCO_2_ between 35 and 45mmHg
**B. Interruption of mechanical ventilation with oxygen supplementation**
B1. Conventional method
- Transtracheal catheter at the level of the carina with oxygen flow rate of 6 L/minute
- T-tube connected to orotracheal tube with oxygen flow rate of 12 L/minute
B2. Application of CPAP
- CPAP valve set at 10cmH_2_O + oxygen flow rate of 12L/minute
- Specific ventilator for noninvasive ventilation. CPAP at 10cmH_2_O + oxygen flow rate of 12L/minute
- Mechanical ventilator in use, adjusting CPAP to 10cmH_2_O + 100% FiO_2_
**C. Confirmation of apnea**
- Lack of spontaneous breathing movements after maximal stimulation of the respiratory center
- 8 - 10 minutes is usually sufficient to obtain PaCO_2_ > 55mmHg
- Obtain final blood gas analysis
**D. Interruption of the test**
- Interrupt the test if there is an arrhythmia, SatO_2_ < 85% or below the limits for the age group.
- Collect blood for blood gas analysis at the time of interruption, even with a shorter observation time
**E. Interpretation**
- Positive test: absence of breathing movements and PaCO_2_ > 55mmHg
- Inconclusive test: absence of breathing movements and PaCO_2_ ≤ 55mmHg
- Negative test: detection of breathing movements after ventilation is discontinued

SatO_2_ - saturação arterial de oxigênio; FiO_2_ - fração inspirada de oxigênio; PaO_2_ - pressão arterial de oxigênio; PaCO_2_ - pressão parcial de dióxido de carbono; CPAP - pressão contínua nas vias aéreas.
  - In cases where the incapacity of oxygenation prevents the disconnection of the mechanical ventilator, the application of **continuous positive airway pressure (CPAP) is provided**.
  - The apnea test is positive for BD when a lack of spontaneous breathing movements is observed with maximal stimulation of the respiratory center with **PaCO_2_ > 55mmHg**.
  - To ensure the safety of the procedure, **the test should be interrupted immediately if there is severe clinical instability** (severe hypotension, severe hypoxemia and cardiac arrhythmia) **or spontaneous breathing**.
- Complementary tests are still required in all cases, and the tests that can be used for diagnosis are cerebral angiography, transcranial Doppler ultrasound, brain scintigraphy and electroencephalogram.^(5)^

## TRAINING FOR THE DIAGNOSIS OF BRAIN DEATH

According to CFM resolution 2,173/2017, **physicians with at least 1 year of experience in the care of comatose** patients and who meet one of the following two criteria are considered **qualified** to perform the clinical examination for diagnosing BD.^(5)^

1. **Have performed or participated in ten diagnoses of BD.**
2. **Have participated in a training course for BD diagnosis.**

## COMMUNICATION AND WITHDRAWAL OF LIFE SUPPORT

- **Appropriate communication of the process of BD diagnosis to family members** is addressed explicitly in both the new resolution and in the Presidential Decree.^(4,5)^ Clear communication must be provided throughout the process by the care team, from the moment of suspicion of BD to the confirmation of death, and must be documented in the medical record. Discussions about organ donation should not occur before the diagnosis of death. It is recommended that the interview with the family regarding organ donation be conducted by a professional with specific training on this topic.
- **The withdrawal of life support** was already contemplated in CFM Resolution 1,826/2007.^(6)^ Now, both CFM Resolution 2,173/2017 and Presidential Decree 9,175/2017, which regulates the Organ Transplantation Law of 1997, define the legality of **discontinuing life support when organ donation is not feasible**, at which point the body must be delivered to the family or sent for an autopsy.^(2,4,5)^

## DOCUMENTATION

The determination of BD is a legal act that must be **documented by the physician in the Statement of Diagnosis of BD (TDME - *Termo de Declaração de ME*), in the medical records and in the Death Certificate**.^(5)^ There has been a significant change in the new TDME. **The complete and appropriate filling of this document is the responsibility of the medical team that performed the diagnostic procedures**. The data must match the information in the medical records and the laboratory reports to ensure that all physiological prerequisites have been met. The requirement of a TDME for all cases favors the exercise of the right to the diagnosis of BD for each citizen and helps obtain more reliable information on the epidemiology of BD in the country. In addition to the TDME, all stages of diagnosis and all communication with relatives should be documented in the medical records. The death certificate should be completed by one of the physicians who established the diagnosis of BD or by assistant physicians or their substitutes. In cases of death from external causes (accident, suicide or homicide), confirmed or suspected, the death certificate is the responsibility of the coroner. The date and time of death correspond to the time of the conclusion of the last procedure for the determination of BD.

### Strong points

A series of changes proposed by CFM Resolution 2,173/2017 help to speed up the diagnosis of BD without compromising safety.

The express definition of **physiological prerequisites** and a minimum time of observation (Table 2) of patients before beginning the protocol for BD diagnosis are recommended in different countries because they provide safety and credibility to the process.

**The nonmandatory participation of a neurologist** in the diagnosis is also in line with several international guidelines. In the United Kingdom, the participation of a neurologist as a consultant to the team that makes the diagnosis is suggested in cases of greater complexity. On the other hand, the **specific qualification** of physicians responsible for the diagnosis of BD is recommended or suggested in different countries to increase the safety of the procedure.^(7-11)^

**The reduction in the time interval between the first and the second clinical examinations** is consistent with the current practices defined in different international guidelines for the determination of BD. In Canada, two physicians are responsible for conducting a clinical examination;^(5)^ in Australia, New Zealand^(8)^ and the United Kingdom,^(9)^ two clinical examinations are performed by two physicians, but without a predetermined time interval; in the United States, a single examination is performed by a physician.^(10)^

**The possibility of establishing the diagnosis of BD in patients with congenital or acquired unilateral anatomical lesions of the eyes or ears** allows, as in other countries,^(7-11)^ the diagnosis of BD in a significant portion of individuals, which was previously not possible in Brazil. The safety of the diagnosis is not affected by this recommendation, considering the requirement for a complementary test in all cases, in addition to the performance of two clinical examinations by two different physicians. In countries where the complementary test is optional, it is used as an alternative or "substitute" for the untested reflex.^(7-11)^

**The reduction from two apnea tests to only one** provides both greater speed and greater safety because it reduces by half the exposure to the risks inherent to this procedure. Performing only one test is common practice in different countries, regardless of the number of recommended clinical examinations.^(7-11)^

The completion of **the complementary test** is optional according to most international guidelines because the essence of the diagnosis of BD is considered to be clinical.^(7-11)^ In Brazil, however, the complementary test is mandatory.^(5)^ This requirement ensures the safety of the diagnosis and appears to be especially beneficial in cases in which there is a possibility of confounding factors, such as exogenous intoxication, the use of central nervous system depressant drugs or metabolic changes caused by liver or kidney failure.

### Controversial points

CFM Resolution 2,173/2017 represents major advances. However, some aspects deserve to be debated due to their possible technical, ethical and social implications.

In some countries where the complementary test is optional, the test is chosen to be performed in order to preclude confounding factors or even to replace a portion of the examination that is not feasible (e.g.: lack of both eyes, bilateral tympanic injury, impossibility to complete the apnea test). Considering that the test is mandatory in Brazil, its use for replacing not feasible phases could be a theme for future debate.

**The resolution originally defined the requirement for PaO_2_ ≥ 200mmHg and PaCO_2_ between 35 and 45mmHg** before starting the apnea test. Consistent with the pathophysiological rationale and guidelines for the diagnosis of BD, the CFM Technical Council rectifies the wording of the original text, recommending that the target values in blood gasses (PaO_2_ ≥ 200mmHg and PaCO_2_ between 35 and 45mmHg) **should, but are not required to**, be met prior to the apnea test after ventilation with 100% oxygen for 10 minutes. Therefore, in cases of severe lung injury, alternatives to the apnea test (CPAP, recruitment maneuvers and aspiration of tracheal secretions) are suggested as long as all the prerequisites are met (Table 3).^(5)^

**Cerebral angiotomography** is another relevant aspect. Although the method was already being used in some Brazilian hospitals, and two recent systematic reviews have concluded that cerebral angiotomography can be used as a complement to the clinical examination for the diagnosis of BD, there is no international standardization of the radiological criteria for the diagnosis of BD. Without the standardization of criteria, sensitivity can vary between 52% and 100%. On the other hand, standardization of the method results in a sensitivity of 87.5%, which implies some risk of false negatives, but without false positives. The greatest limitation of the existing studies is the fact that it was not possible to assess specificity due to the lack of false positives (all patients were brain dead). Future randomized clinical trials may lead to a reassessment of this parameter.^(12,13)^

Finally, CFM Resolution 2,173/2017 defines parameters to standardize the legal definition of BD and innovates by incorporating a Manual of Procedures for Diagnosis of Brain Death (in its Appendix I), which provides guidelines on the methodology to be used for the diagnosis of BD. The manual is linked to the body of the resolution and determines, with the force of law, the medical procedures to be used for the diagnosis of BD.^(5)^

In the United States, in contrast, a presidential commission established a legal framework only to standardize the definition of BD (the equivalent of the body of resolution 2,173/2017) but without restricting the "standard of medical procedures" to be used for such diagnosis. The definition of such standards is the responsibility of a group of specialists from the American Academy of Neurology (AAN) that develops care guidelines (guidance tools and not legal instruments) based on a careful review of the available evidence, using data searches and interpretation techniques in accordance with the methods required by evidence-based medicine.^(5,10)^

This seems to be where the greatest weakness of resolution 2,173/2017 lies. The legal nature of the technical manual limits the possibilities for the diagnosis of BD and precludes the use of available resources and new resources, even if supported by the technical literature on the subject.^(14)^

## FINAL COMMENTS

The new resolution that establishes the criteria for the diagnosis of BD published by the CFM represents an advance in ensuring the safety of this diagnosis in our country and is in line with medical scientific advances. It enables the training of professionals from specialties that, in their day to day practices, provide care for critically ill patients in coma and is based on well-established criteria and in agreement with international guidelines. Thus, in addition to patient safety, the CFM promotes the reinforcement of its important social role and the role of all physicians in the preservation of life through organ donation and transplants. Similarly, it highlights the importance of the involvement and the key role of medical associations, such as the *Associação de Medicina Intensiva Brasileira* (AMIB), the *Academia Brasileira de Neurologia* (ABN), the *Colégio Brasileiro de Radiologia* (CBR) and the *Associação Brasileira de Medicina de Emergência* (ABRAMEDE), for the dissemination of information on the topic and the specific training of Brazilian physicians for the safe determination of BD.
